# Medium- and large-sized mammals from Estação Biológica Fiocruz Mata Atlântica, Rio de Janeiro, south-eastern Brazil

**DOI:** 10.3897/BDJ.10.e86756

**Published:** 2022-07-25

**Authors:** Iuri Veríssimo, Gabriel Cupolillo, Beatriz Maria da Silva Jorge, Roberto Leonan Morim Novaes, Jonatas Amorim Tavares, Monique Medeiros Gabriel, Sócrates Fraga Costa-Neto, Ademar Luiz Gomes do Couto, Ellen Schmidt, Amarildo Miranda, Cecilia Siliansky de Andreazzi, Ricardo Moratelli

**Affiliations:** 1 Fiocruz Mata Atlântica, Fundação Oswaldo Cruz, Rio de Janeiro, Brazil Fiocruz Mata Atlântica, Fundação Oswaldo Cruz Rio de Janeiro Brazil; 2 Programa de Pós-Graduação em Ecologia, Instituto de Biologia, Universidade Federal do Rio de Janeiro, Rio de Janeiro, Brazil Programa de Pós-Graduação em Ecologia, Instituto de Biologia, Universidade Federal do Rio de Janeiro Rio de Janeiro Brazil; 3 Programa de Pós-graduação em Biodiversidade e saúde, Instituto Oswaldo Cruz, Fundação Oswaldo Cruz, Rio de Janeiro, Brazil Programa de Pós-graduação em Biodiversidade e saúde, Instituto Oswaldo Cruz, Fundação Oswaldo Cruz Rio de Janeiro Brazil; 4 Laboratório de Biologia e Parasitologia de Mamíferos Silvestres Reservatórios, Instituto Oswaldo Cruz, Fundação Oswaldo Cruz, Rio de Janeiro, Brazil Laboratório de Biologia e Parasitologia de Mamíferos Silvestres Reservatórios, Instituto Oswaldo Cruz, Fundação Oswaldo Cruz Rio de Janeiro Brazil; 5 Centro de Ecología Funcional, Universidade de Coimbra, Coimbra, Portugal Centro de Ecología Funcional, Universidade de Coimbra Coimbra Portugal

**Keywords:** camera traps, conservation, diversity, domestic dog, habitat use, Pedra Branca Forest, species richness

## Abstract

The Pedra Branca Forest is in a highly urbanised region of the central portion of Rio de Janeiro City and comprises the largest urban forest in the world (> 12,000 ha). The local flora and fauna are protected by three conservation units and the Estação Biológica Fiocruz Mata Atlântica (EFMA), which comprises 462 hectares on the east side of the remnant. The local biodiversity is still little known compared to other Atlantic Forest remnants from the Rio de Janeiro State. Here, we provide results of a survey of medium- and large-sized terrestrial mammals from the EFMA. In addition, we analysed the distribution of this fauna along three habitat types defined as Peridomicile, Transitional Forest and Forest Core. Sampling was performed from 2017 to 2020 and comprised a camera-trap survey, interviews with residents and local workers and occasional records. Results include occurrence records for 16 autochthonous and one allochthonous (*Callithrix* sp.) wild mammals, which are distributed into 14 families and seven orders, in addition to the presence of free-ranging domestic dogs and cats. Four species are in some category of threat of extinction at national or global levels. Amongst them, *Leontopithecusrosalia* (first record for the Rio de Janeiro City in more than a century) and *Leopardusguttulus* are classified as Vulnerable by IUCN. Most wild native species were registered in the three habitat types, but with differences in the frequency of records. Our results indicate that the presence of domestic dogs and cats influenced the species composition in each area, with *Nasuanasua*, *Dasyproctaleporina* and *Didelphisaurita* less frequent in places where domestic dogs and cats are more frequent. This is the first systematic effort to understand the occurrence and distribution of mid- and large-sized mammals in the Pedra Branca Forest.

## Introduction

Three Atlantic Forest remnants—Pedra Branca, Tijuca and Gericinó-Mendanha—are present in Rio de Janeiro City. The Pedra Branca Forest is the largest remnant of urban forest in the world and is located in a highly urbanised region of the central portion of the City. This remnant is partially connected to the Tijuca Forest by small forest fragments separated by highways and both are isolated from the Gericinó-Mendanha Forest by a matrix of urban densification. The flora and fauna of Pedra Branca are protected by the Parque Estadual da Pedra Branca (PEPB; Pedra Branca State Park), Parque Natural Municipal da Prainha, Reserva Biológica de Guaratiba and the Estação Biológica Fiocruz Mata Atlântica (EFMA; Fiocruz Atlantic Forest Biological Station). Most of the territory is preserved by the PEPB, which comprises areas above 100 m a.s.l. (ca. 12,000 hectares). The EFMA is on the east side of the remnant, in an area under high human pressure, whose biological diversity, including mammals, is still poorly known compared to other localities in the Municipality and in the State of Rio de Janeiro—for example, Tijuca Forest (Freitas et al. 2006, Silva 2017, Silva et al. 2018) and Serra dos Órgãos (Cronemberger et al. 2019), respectively.

The EFMA is adjacent to six communities with high social vulnerability and precarious sanitation conditions. These communities extend through the forest edge, putting domestic and wild animals, insect vectors and humans in potential contact, which constitutes a favourable environment for the circulation of zoonotic and non-zoonotic pathogens (White and Razgour 2020). From the viewpoint of wildlife surveillance, the scenario deserves special attention, since outbreaks of emerging and re-emerging infectious zoonotic diseases are associated with interactions between pathogens and potential hosts (usually mammals) and anthropogenic changes in the environment, including habitat loss, socioeconomic factors and demographic increase (Daszak et al. 2012, Jones et al. 2008).

Studies with mammals in the Pedra Branca Forest that used systematised sampling were only conducted for the small-sized species, including bats, rodents and marsupials (Gentile et al. 2018, Tavares et al. 2021). Thus, the few records of mid- and large-sized mammals that exist for this region are the result of occasional observations. We carried out a survey of medium and large-sized mammals and analysed the distribution of this fauna from the peridomicile to the forest core, as part of a project to understand the ecological interfaces that may favour the circulation of zoonotic and non-zoonotic pathogens amongst humans, wildlife and domestic animals in the EFMA territory.

## Data resources

Individualised records of medium- and large-sized mammals from Fiocruz Atlantic Forest Biological Station, Rio de Janeiro, south-eastern Brazil, registered by camera trap, are available in Suppl. material 1. Results from SIMPER analysis with the contribution of each species to overall dissimilarities amongst sampling areas are available in Suppl. material 2.

## Material and Methods

### Study area

**Figure 1.**
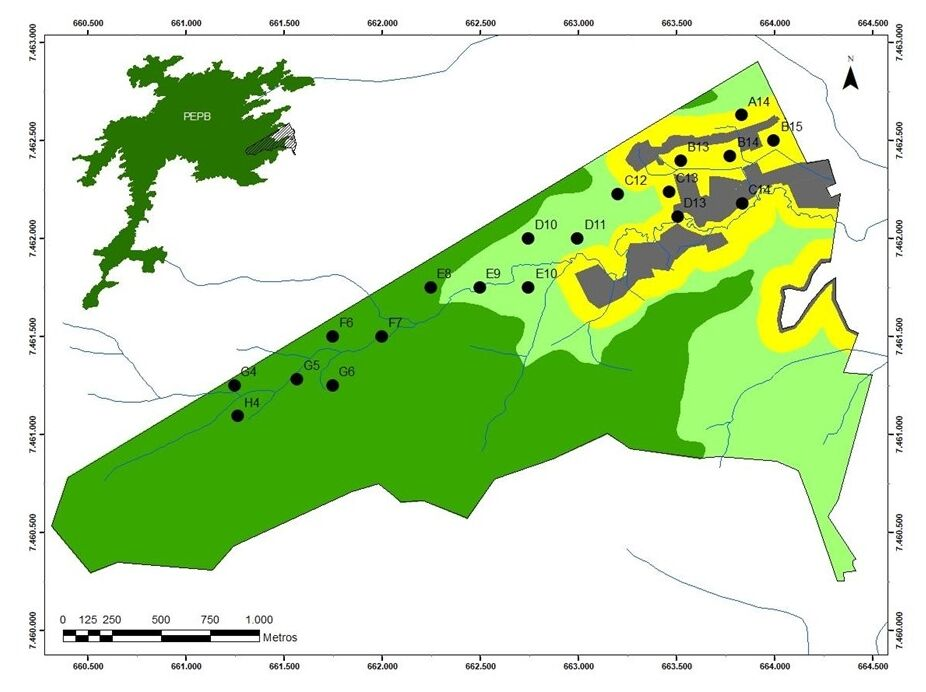
The location of the Estação Biológica Fiocruz Mata Atlântica (EFMA) on the east side of Pedra Branca Forest (left, above), Rio de Janeiro; and the distribution of camera-traps at EFMA, in areas defined as Peridomicile (yellow), Transition Forest (light green) and Forest Core (dark green). Combinations of letters and numbers refer to camera identifications. See Table 1 for coordinates, period and effort at each point.

The study was carried out at the Estação Biológica Fiocruz Mata Atlântica (EFMA; central coordinates 22°56'25" S, 43°24'18" W, Fig. 1), located on the eastern slope of the Pedra Branca Forest, Municipality of Rio de Janeiro, Brazil. The EFMA comprises 462 ha, of which 262 ha (57%) overlap with the Parque Estadual da Pedra Branca (PEPB). It is made up of remnants of both the Lowland Dense Ombrophilous Forest (50 m a.s.l.) and the Submontane Dense Ombrophilous Forest (50–500 m a.s.l.). The lowland forest is composed of different habitat types impacted by the presence of six communities with high social vulnerability and precarious sanitation conditions. These communities are connected at different levels to densely populated neighbourhoods in the Jacarepaguá region. In addition to a high demographic occupation, other anthropogenic impacts are present, such as small-scale agricultural and poaching activities (Domingues and Rodrigues 2007).

The areas used for the mammal survey are distributed along a gradient of anthropogenic intervention, where each area defined for sampling represents a type of habitat along this gradient: (i) Peridomicile, which consists of areas up to 100 m adjacent to the communities (ca. 30–35 m a.s.l.) and is characterised by the presence of backyards and orchards, with vegetation dominated by exotic species; (ii) Transitional Forest, which extends from the end of the peridomicile area to 100 m.a.s.l., with a prevalence of native plants, but with dense understorey and low canopy; and (iii) Forest Core, including all areas above 100 m a.s.l. and comprising preserved areas, without human constructions, with little human activities, more open understorey, high canopy and great presence of epiphytic plants, streams and rock formations on a sloping topography.

#### Sampling design and data collection

The species survey was conducted using three different methods: (i) sampling with camera-traps; (ii) interviews with residents who live around the EFMA; and (iii) occasional records made directly by researchers during field activities. Only mammals whose species-level identification can be performed, based on external morphology, were considered in the results. This procedure allowed the inclusion of all mammals with a body weight of > 1.0 kg (Chiarello 2008), as well as representatives of other taxa that could be reliably identified in the sampled area (e.g. *Didelphis*, *Sylvilagus*). The nomenclature used for xenarthrans and marsupials followed different authors in Gardner (2008). For the others, the nomenclature follows Wilson and Reeder (2005).

For the camera trap survey, Trophy Cam trail cameras (Bushnell, Overland Parks, KS, USA) were distributed at 19 points along the areas defined as peridomicile, transitional forest and forest core (Table 1, Fig. 1). The sampling grid followed a minimum distance of 250 m between the points, including the altitudinal gradient, covering much of the environmental heterogeneity of the EFMA. The study was conducted from June 2018 to May 2020, with one camera positioned at each point (Table 1). These cameras were installed ca. 40 cm above the ground. They were installed randomly in spots where animals are expected to pass, such as trails, forest clearings and near fruit trees. Camera traps remained operational from 17 to 306 days throughout the study period and were checked every 30 days to change memory cards and batteries when needed. The images of all individuals of the same species detected by the same camera trap within a 1-h interval were treated as a single record.

The interviews to survey the species that occur in the region were conducted with residents, Fiocruz employees involved in the maintenance of EFMA trails and researchers who work with the local fauna and flora. Occasional records of mammals made by our staff or by other Fiocruz researchers were also considered.

#### Data analyses

Effort for the camera trap survey was calculated by multiplying the number of cameras installed by the number of days remaining active (unit: camera-days; Srbek-Araujo and Chiarello 2007), totalling 2,683 camera-days. Sampling sufficiency was verified by the rarefaction curve (Mao Tau) of the accumulated richness as a function of the days sampled (Soberón and Llorente 1993, Colwell et al. 2004). Estimated richness was calculated using Jackknife-2 and Chao-2 indexes (Zahl 1977, Chao 1984). An Analysis of Variance (ANOVA) was used to test the statistical significance of differences between the averages of the records/camera-days between the sampling areas in the EFMA. The Shapiro-Wilk test was used to confirm the normal distribution of the data. These analyses were all performed using PAST 4.01 software.

The homogeneity of multivariate dispersions was tested following Anderson (2006). We explored the patterns of similarity of records between areas using multivariate permutational analysis of variance (PERMANOVA) and non-metric multidimensional scaling analysis (nMDS) on Jaccard distances from the presence and absence matrix. Average similarity amongst sampling points was achieved by performing a Hierarchical agglomerative clustering using Ward's method and cluster adequacy was accessed through a cluster-wise cluster stability assessment by bootstrap resampling (Hennig 2007). A similarity percentage (SIMPER) analysis was performed to compare the contributions of the species between sampling sites. SIMPER analysis is based on the Bray-Curtis Index for estimating the average dissimilarity between pairs of sample groups and determining the contributions of each species to the average between-group Bray-Curtis dissimilarity (Clarke 1993). To assess whether the presence of records of domestic dogs and cats influenced the composition of wild animal records, we also used PERMANOVA to test differences between the sampling points with and without the domestic species, considering only the matrix of presence and absence of wild species. All analyses were performed using the “vegan”, "cluster" and "fpc" packages in the R platform (Oksanen et al. 2020, R Core Team 2021).

The conservation status of each species on a global and national level was derived respectively from the IUCN Red List of Threatened Species (version 2021.3) and the Red Book of Threatened Brazilian Fauna (Instituto Chico Mendes de Conservação da Biodiversidade 2018). We also report the status of each species in the list of the Convention on International Trade in Endangered Species of Wild Fauna and Flora (Convention on International Trade in Endangered Species of Wild Fauna and Flora 2021).

## Results

In addition to domestic dogs and cats, the camera trap survey, interviews and other sporadic records revealed the presence of 17 species of medium- and large-sized autochthonous (16 spp.) and allochthonous (*Callithrix* sp.) wild mammals, which are organised into 14 families and seven orders (Table 2).

The sampling effort with camera-traps provided 1,189 records of 12 species of wild mammals and domestic dogs and cats. Eighteen species were recorded by interviews, of which nine were not recorded by camera traps (*Bradypusvariegatus*, *Cabassoustatouay*, *Dasypusseptemcinctus*, *Dicotylestajacu*, *Eirabarbara*, *Euphractussexcinctus*, *Galictiscuja*, *Leontopithecusrosalia* and *Sapajusnigritus*). Amongst them, the following six were included in our list: *B.variegatus*, *Leontopithecusrosalia*, *S.nigritus* and *T.tetradactyla*, which were confirmed by direct observation; and *C.tatouay* and *E.sexcinctus*, whose occurrence is confirmed for Tijuca Forest—an Atlantic Forest remnant geographically close to EFMA. Although the occurrence of *D.tajacu* has been confirmed for the Mendanha Forest—another Atlantic Forest remnant geographically close to EFMA—we chose not to include it in our list due to the rarity of the species in the Municipality of Rio de Janeiro. Thus, the records of *Eirabarbara*, *Galictiscuja*, *Dicotylestajacu* and *Dasypusseptemcinctus* obtained from interviews were considered dubious. Eight species were recorded by direct observation, none exclusively.

Of the 17 wild mammal species recorded, all are autochthonous, except *Callithrix* sp., which is a hybrid of *Callithrixjacchus* and *Callithrixpenicillata*, widely distributed in the City of Rio de Janeiro. The record of *Leontopithecusrosalia* is the first of the current distribution of the species to the Municipality of Rio de Janeiro. *Leontopithecusrosalia* is coded as Endangered in the Red Book of Threatened Brazilian Fauna (Instituto Chico Mendes de Conservação da Biodiversidade 2018) and IUCN (Ruiz-Miranda et al. 2015). *Leopardusguttulus* is classified as Vulnerable in the Red Book of Threatened Brazilian Fauna (Instituto Chico Mendes de Conservação da Biodiversidade 2018) and IUCN (Oliveira et al. 2014). *Sapajusnigritus* is coded as Near Threatened in the Red Book of Threatened Brazilian Fauna (Instituto Chico Mendes de Conservação da Biodiversidade 2018), but the species has not been evaluated by the IUCN. *Leontopithecusrosalia* and *Leopardusguttulus* are included in Appendix I of CITES; *Bradypusvariegatus* and *Cerdocyonthous* are in Appendix II; and *Cuniculuspaca* is listed in Appendix III of CITES.

Considering only the results of camera-traps, the highest concentration of records occurred in the peridomicile (*N* = 519, 0.391/camera-days), followed by the forest core (*N* = 375, 0.472/camera-days) and transitional forest (*N* = 295, 0.525/camera-days; Table 3). Of 12 species recorded by camera-traps, 10 occurred in the Peridomicile and Transitional Forest and nine in the Forest Core. The most recorded species in camera-traps was *Didelphisaurita* (*N* = 370, 0.138/camera-days), followed by *Cuniculuspaca* (*N* = 279, 0.104/camera-days), *Canislupusfamiliaris* (*N* = 174, 0.065/camera-days) and *Dasyproctaleporina* (*N* = 164, 0.065/camera-days; Table 3).

In the peridomicile, the most frequent species were *Cuniculuspaca* (*N* = 162, 0.122/camera-days), *Didelphisaurita* (*N* = 146, 0.110/camera-days) and *Canislupusfamiliaris* (*N* = 122, 0.092/camera-days); in the transitional forest, were *Didelphisaurita* (*N* = 166, 0.295/camera-days), *Dasyproctaleporina* (*N* = 66 records, 0.117/camera-days) and *Canislupusfamiliaris* (*N* = 21, 0.037/camera days); while in the forest core, the predominant species were *Cuniculuspaca* (*N* = 111, 0.140/camera-days), *Dasyproctaleporina* (*N* = 97, 0.122/camera-days) and *Didelphisaurita* (*N* = 58, 0.073/camera-days; Table 3).

*Didelphisaurita* showed the highest frequency of occurrence, found in 89% of the sampling points, followed by *Canislupusfamiliaris*, with 63%, and *Tamanduatetradactyla* with 58%. The domestic dog was recorded at all peridomicile points, in addition to records in some transitional forest and forest core points. However, domestic cats, *Feliscatus* and *Sylvilagustapetillus* were only found in the peridomicile points. On the other hand, *Leopardusguttulus* and *Nasuanasua* were recorded only in the Transitional Forest and Forest Core. *Cerdocyonthous* was recorded in the Peridomicile and Transitional Forest (Table 4).

**Figure 2.**
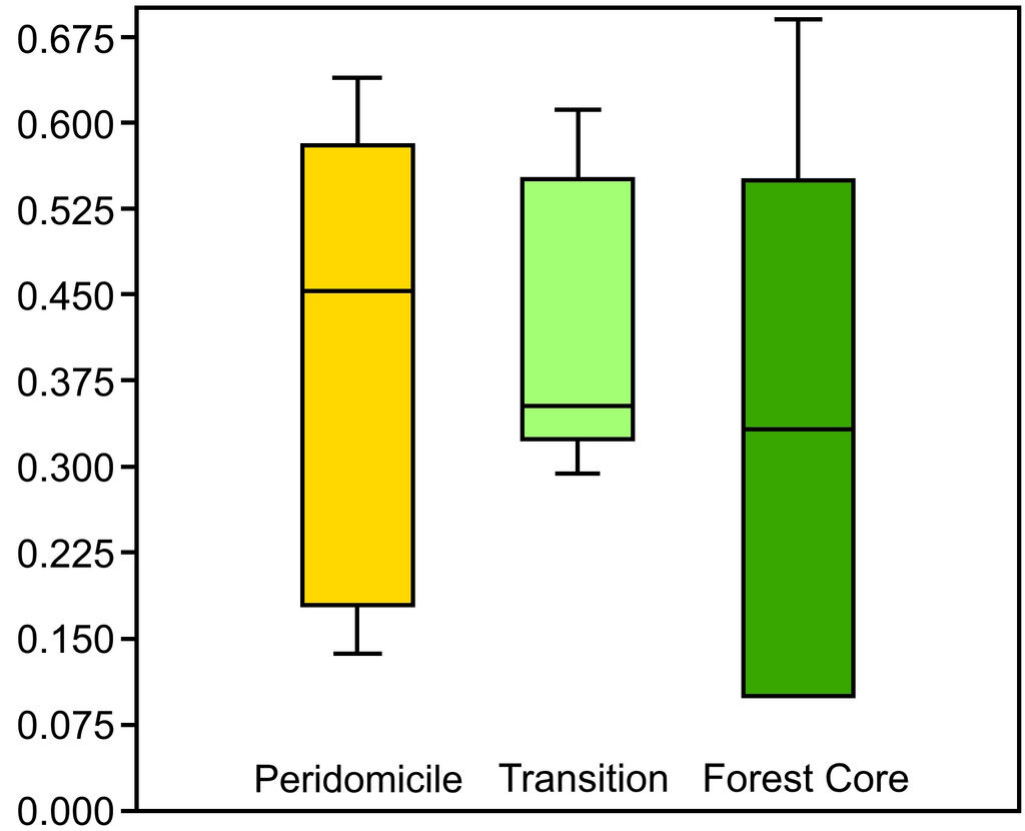
Analysis of variance (ANOVA) for records/camera-days for each sampling area (Peridomicile, Transitional Forest, Forest Core) for mid- and large-sized mammals from Estação Biológica Fiocruz Mata Atlântica, Rio de Janeiro.

**Figure 3.**
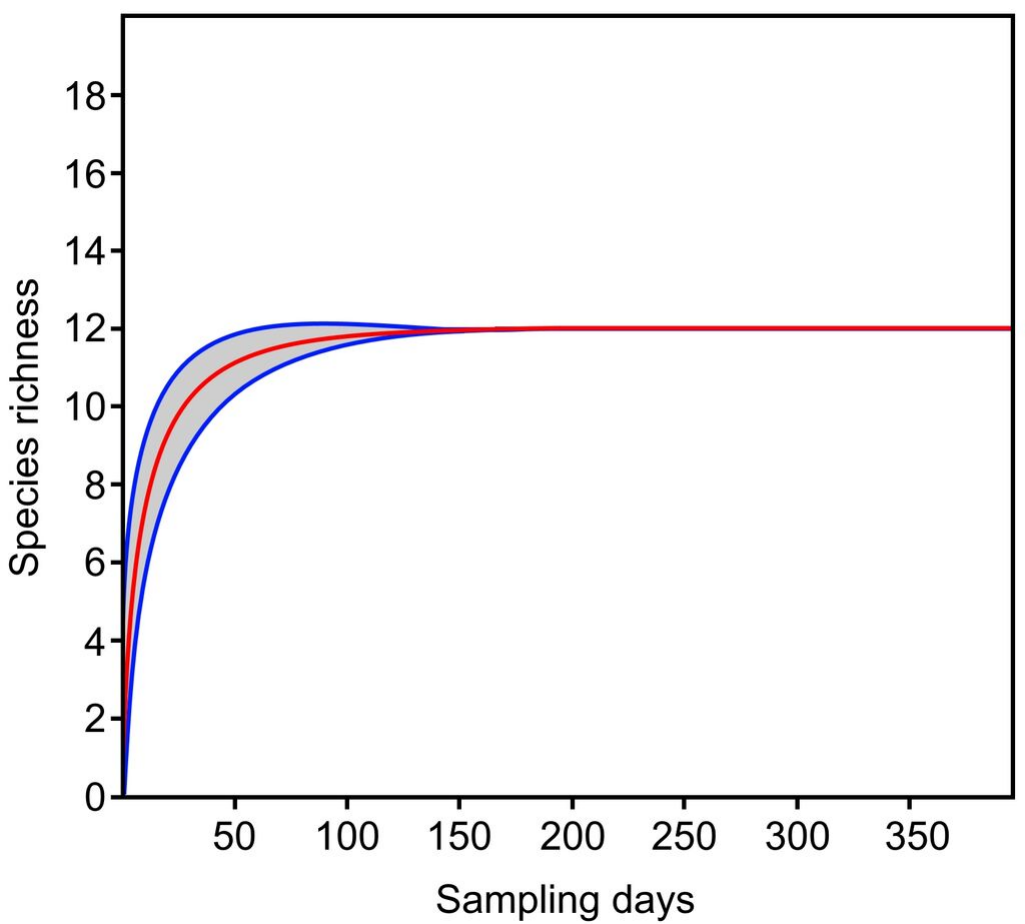
Rarefaction curve (Mao Tao) of accumulated richness by sampling days for mid- and large-sized mammals from Estação Biológica Fiocruz Mata Atlântica, Rio de Janeiro. Blue lines correspond to the 95% confidence interval.

Considering the three sampling areas (Peridomicile, Transitional Forest and Forest Core) as independent populations, we did not find significant differences between the averages of camera-day records for these areas (*F* = 0.238; *p* = 0.792; Fig. 2). The Shapiro-Wilk test indicated the normality of the data (*p* = 0.949). The rarefaction curve (Mao Tao) of the accumulated richness by the sampling days reached its asymptote with 12 species recorded between 100 and 150 sampling days (from a total of 395 days; Fig. 3). The observed richness was the same as estimated by the Jackknife-2 and Chao-2, within the 95% confidence interval of the observed species.

**Figure 4.**
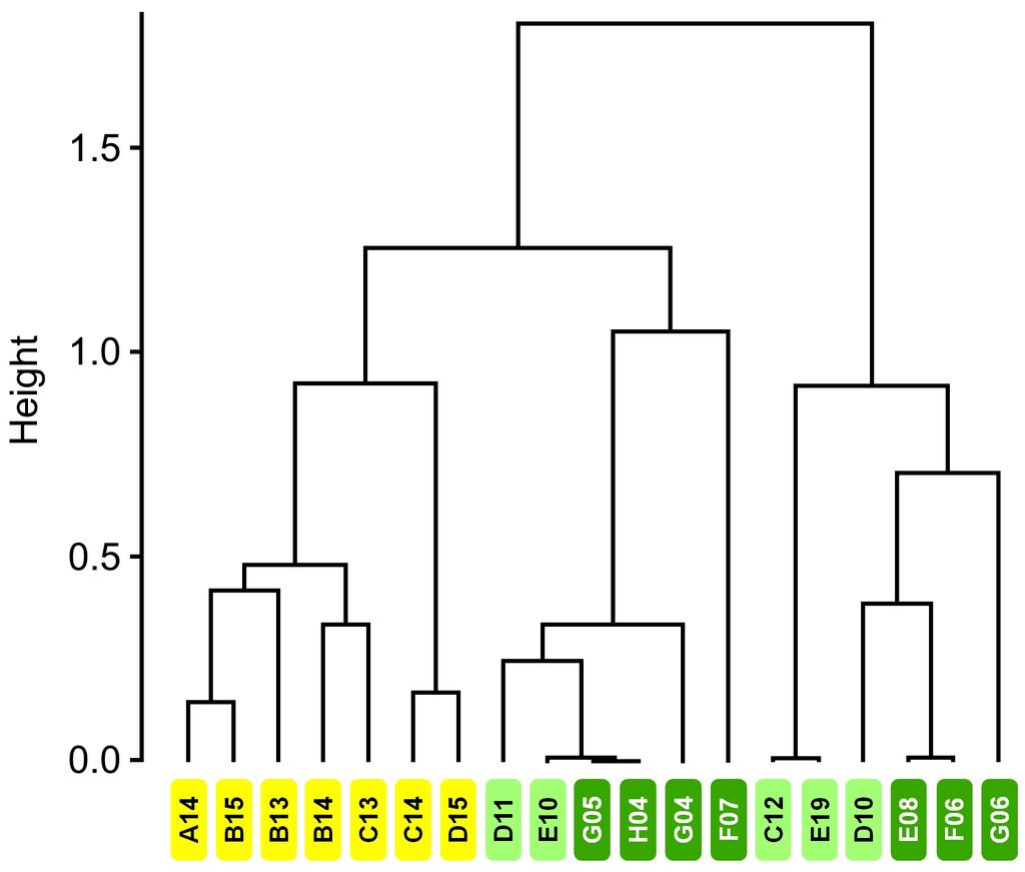
Dendrogram produced by cluster analysis (Ward method) from the similarity (Jaccard distance) between points in the peridomicile (yellow), transitional forest (light green) and forest core (dark green), considering the species richness of medium and large-sized mammals in the Estação Biológica Fiocruz Mata Atlântica, Rio de Janeiro.

**Figure 5.**
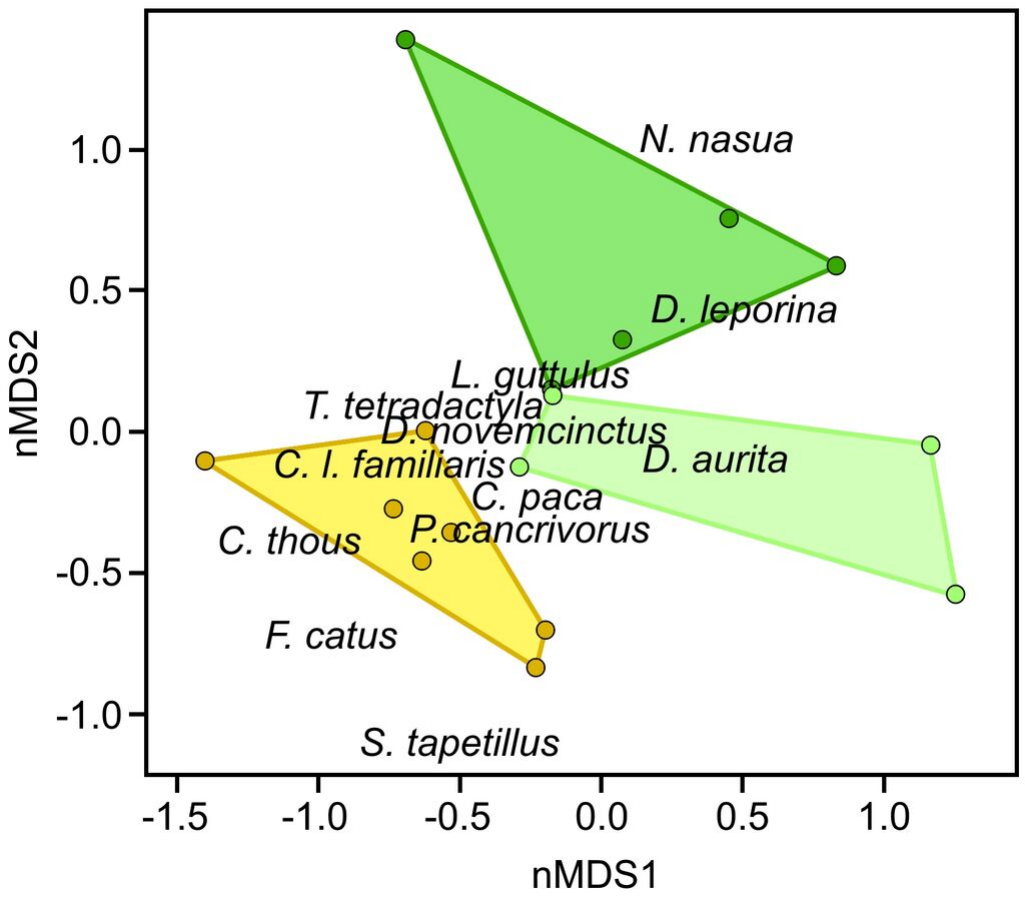
Plot of the nMDS analysis, based on Jaccard distances showing sampling points and species coordinates. The convex polygons delimit the peridomicile (yellow), transitional forest (light green) and forest core (dark green) in the Estação Biológica Fiocruz Mata Atlântica, Rio de Janeiro.

The areas showed homogeneity of multivariate dispersion (*F* = 1.29, *p* = 0.29) and differed significantly in relation to the similarity of the records (*F* = 4.75, *p* < 0.001, Fig. 4). The nMDS analysis (stress = 0.12, r^2^ = 0.98; Fig. 5) revealed no overall overlap between the areas, indicating a difference in the composition of records in each area. In general, most species were recorded in the Peridomicile, but the results indicate that *Nasuanasua* and *Dasyproctaleporina* are more associated with the Forest Core. Indeed, pairwise comparisons of the three areas show that *N.nasua* plays a important role in distinguishing Forest Core from the other two areas (Suppl. material 2). Even though *L.guttulus* was not recorded in Peridomicile, the species was not important to differentiate this area from the others. Still, records of *L.guttulus* significantly differed between the Transitional Forest and the Forest Core (Suppl. material 2). Records of *C.thous* were more abundant in Peridomicile and SIMPER analyses showed its importance in differentiating this area from the other two (Suppl. material 2). *Didelphisaurita* was recorded in almost all points, but with a highest concentration in the Transitional Forest. Considering only wild species, our results revealed that the presence of domestic dogs (*F* = 5.14, *p* = 0.001) and domestic cats (*F* = 3.25, *p* = 0.008) influenced species composition, with *Nasuanasua*, *Dasyproctaleporina* and *Didelphisaurita* less frequently registered in the points where domestic dogs and cats were most frequent. Additionally, records of domestic animals were important to differentiate Peridomicile from Transitional Forest and Forest Core (Suppl. material 2) with a higher number of records for these species near to more anthropogenised areas.

Our data indicated the existence of three stable groups (mean bootstrapped Jaccard similarities > 0.85 and overall cluster instability < 0.06 for all three clusters). Peridomicile records clearly differentiate this area from the other two (i.e. Transitional Forest and Forest Core) as all sample points defined as Peridomicile were grouped into a single cluster (Fig. 4). The other two groups included points from both Transitional Forest and Forest Core and this analysis did not allow the differentiation of these two areas (Fig. 4).

## Discussion

### Species richness

About 60 spp. of mammals from different orders are known to occur in the region of the Pedra Branca Forest (Secretaria do Meio Ambiente 2013, Gentile et al. 2018, Pontes et al. 2021, Tavares et al. 2021). However, standardised efforts were focused on bats, rodents and marsupials (Gentile et al. 2018, Tavares et al. 2021). Records of medium- and large-sized mammals were based on occasional observations. Thus, this is the first standardised sampling effort to survey medium and large mammals in the region. To EFMA, we considered valid the records of 17 species of autochthonous and allochthonous wild mammals distributed in 14 families and seven orders, in addition to domestic dogs and cats. All species recorded in EFMA are known to occur in other protected areas in the State of Rio de Janeiro (e.g. Modesto et al. 2008, Delciellos et al. 2012, Silva 2017, Silva et al. 2018). In the Parque Nacional da Tijuca, which is the geographically closest Atlantic Forest remnant to EFMA, Silva et al. (2018) recorded 16 species of medium and large mammals, with the dominance of *Nasuanasua*, *Didelphisaurita* and *Cuniculuspaca*. Some species, registered in the Tijuca Forest using camera traps, such as *Cabassoustatouay* and *Sapajusnigritus*, were not recorded by this method in the EFMA, but were included in the list, based on the results of interviews (*Cabassoustatouay* and *Sapajusnigritus*) and subsequent observation (*Sapajusnigritus*). Recently, Pontes et al. (2021) recorded the occurrence of *Pumaconcolor* on the west side of Pedra Branca Forest and surroundings. Despite the rarity of the species in the metropolitan region of Rio de Janeiro, we do not rule out the possibility of its occurrence within the limits of the EFMA, considering its connectivity with the rest of the remnant.

Despite differences in habitat structure and composition amongst the Peridomicile area, Transitional Forest and Forest Core, differences were not observed in the abundance of medium and large mammals amongst these areas in the EFMA. The survey focused mainly on the use of camera traps because they favour records of species that are difficult to detect through active search and because the method requires less effort when compared to active search or capture (Carbone et al. 2001, Santos-Filho and Silva 2002, Silveira et al. 2003, Trolle 2003, Srbek-Araujo and Chiarello 2005). Surveys of mid- and large-sized mammals using camera traps have already been carried out in several protected areas in the State of Rio de Janeiro, including Parque Nacional da Tijuca (Silva et al. 2018), Parque Nacional da Restinga de Jurubatiba (Xavier 2016), Parque Nacional de Itatiaia, Parque Nacional da Serra dos Órgãos (Aximoff et al. 2015), Reserva Ecológica de Guapiaçu (Carvalho et al. 2014), Parque Nacional da Serra da Bocaina (Delciellos et al. 2012), Parque Estadual da Ilha Grande (Lessa 2012) and Parque Estadual do Desengano (Modesto et al. 2008).

In surveys with camera-traps conducted in the mountainous region of Rio de Janeiro, which comprises the largest remnant of continuous Atlantic Forest in the State, more than 20 species of medium and large mammals were recorded, including some threatened, such as *Pumayagouaroundi*, *Pumaconcolor*, *Dicotylestajacu* and *Tayassupecari* (e.g. Carvalho et al. 2014, Aximoff et al. 2015), which are either almost extinct or rare in the Municipality of Rio de Janeiro, such as *Dicotylestajacu* and *Pumaconcolor* (Martins and Pontes 2021, Pontes et al. 2021).

### Conservation remarks

Domestic dogs and cats, which showed high frequency of occurrence in the EFMA, also showed high frequency in Parque Nacional da Tijuca (Silva et al. 2018). The record of domestic animals in protected areas is frequent, especially in metropolitan areas, where the presence of residences in the surroundings or even within these areas is common. According to Silva (2017), the records of domestic dogs in the Tijuca Forest are concentrated during the day, indicating that they are domiciled animals, spending the night in their residences. The activity of dogs in the EFMA is not limited to areas with high human presence (Peridomicile) and overlaps with some species also recorded in the present study, such as *N.nasua* and *D.leporina* (q.v. Silva et al. 2018). The records of dogs in more preserved areas (i.e. Transitional Forest and Forest Core), although at low frequency, agree with findings of Silva et al. (2018). It is important to highlight here that these animals are, in general, domiciled, but raised freely in the territory.

Domestic dogs and cats exert different types of pressure on local biodiversity. Amongst the 17 wild species recorded in the EFMA, there are predation records by dogs for eight species, competition records for six and pathogen transmission reports for two (Lessa et al. 2016). In the Jardim Botânico do Rio de Janeiro, which is adjacent to the Tijuca Forest, Rangel and Neiva (2013) report predation of *D.aurita*, *T.tetradactyla* and *P.cancrivorus*, all recorded at EFMA. *C.thous* is reported as a susceptible species for disease transmission and competition from domestic dogs (Lessa et al. 2016). Our results indicate a significant relationship between domestic dogs and *C.thous* to less preserved areas, thus increasing the risk of those threats emerging in EFMA’s territory. For domestic cats, predation pressure on wild mammals is greater when *Feliscatus* individuals are feral (Oliveira et al. 2010, Loss et al. 2013). The shared land use between domestic cats and wild felid species, such as *L.guttulus* (present in EFMA), is also a threat due to possible niche overlap (Ferreira et al. 2019). Domestic cats might transmit pathogens to wild mammals, such as the rabies virus and pose threats to local wildlife (Gerhold and Jessup 2012). Our results, however, do not indicate the occurrence of domestic and wild cats in the same area, with the records of domestic cats restricted to the Peridomicile and the records of *L.guttulus* in the Transition Forest and Forest Core. *L.guttulus* is commonly reported as a forest dwelling species able to tolerate some degree of human disturbance (Oliveira et al. 2010). Our result is in accordance with this previous report as it shows the relationship of *L.guttulus* to forest areas with intermediate anthropogenic pressure. The diet of *L.guttulus* includes small mammals, even comprising exotic rodent species (Rinaldi et al. 2015, Tortato et al. 2021). High frequencies of synanthropic marsupial and rodent species in anthropogenised areas have already been reported for the area (Gentile et al. 2018), potentially influencing the occurrence of *L.guttulus* in areas with intermediate human disturbance. Results for *N.nasua* are opposed to observations in protected areas that receive tourists, such as Jardim Botânico do Rio de Janeiro (Brazil) and Parque Nacional Iguazú (Argentina) where this species is commonly observed near areas with high human activity (Hirsch 2009, Cunha 2010). Thus, depending on the characteristics of the area, such as tourist visitation and prey abundance, species occurrence and abundance inside an anthropogenic gradient may vary between localities.

In relation to primates, it is also worth mentioning the record of the golden-lion-tamarin, *Leontopithecusrosalia*, in the EFMA and adjacent areas. One individual of this species was recorded by direct observation in 2017, living with a group of *Callithrix*. Subsequently, the species was also reported by residents and Fiocruz employees, who reported the presence of more than one individual, with at least one female and a juvenile. We have records of at least three individuals that co-exist with *Callithrix* in the surroundings of EFMA. The species is endemic to the southeast Atlantic Forest, originally occurring in coastal lowland forests of the States of Rio de Janeiro and southern Espírito Santo (Coimbra-Filho 1969, Kleiman and Rylands 2002). Apparently, in the 1960s, it was already extinct in 17 municipalities, including Rio de Janeiro, remaining restricted to the São João River Basin, with its occurrence limited to the Municipalities of Silva Jardim, Araruama, Cabo Frio and Saquarema (Kierulff 1993). Recently, it was also recorded in forest fragments in the Municipality of Duque de Caxias (Burity et al. 2007). The origin of these individuals in the EFMA and adjacent areas is still uncertain and may be related to the existence of illegal breeding sites in the region.

Peridomicile is a distinct area inside EFMA’s territory concerning medium- large-sized mammal communities, probably by the effects of the high level of anthropogenic disturbances. Habitat loss, introduction of exotic species and poaching are the main threats to mammals in the Pedra Branca Forest (Secretaria do Meio Ambiente 2013). These pressures are the target of mitigation actions in EFMA's territory. Regarding habitat loss, we highlight the control over irregular constructions within the Fiocruz area (Sector 1 of Colônia Juliano Moreira), including the prohibition of new buildings and land expansions. For free-ranging domestic animals, there are actions to raise awareness about responsible custody to reduce the number of unaccompanied animals; inspection actions to reduce the abandonment of animals in the territory; castration and vaccination of abandoned animals; and referral of untrained animals for adoption. These actions to minimise contact between domestic and wild animals aim to reduce predation of wild birds and mammals and minimise the risk of pathogen spill-over from domestic to wild mammals, such as the Canine Distemper Virus (Megid et al. 2013) and the fungus *Sporothrix* spp., which causes sporotrichosis, a zoonotic disease that mainly affects cats, but can also affect humans (Almeida-Paes et al. 2014). Regarding poaching in EFMA, there are campaigns to raise awareness of the legislation that prohibits hunting and articulation with the Environmental Police for actions aimed at curbing the action. These actions apparently reduced the local habitat loss and curbed the poaching activity at EFMA. Despite this, it is still necessary to keep the monitoring of native mammals and develop more research focused on the impact of domestic animals and poaching on the species composition and population dynamics of wild mammals. This was the first study with systematic sampling of the mammals from Pedra Branca Forest and the results are the basis for understanding the ecological interfaces that favour contact between wildlife and humans, directly or indirectly through domestic animals.

## Supplementary Material

DB57CE05-0072-5167-BCF0-134EC6AAB362 10.3897/BDJ.10.e86756.suppl1 Supplementary material 1 Individualised records of mammals from Fiocruz Atlantic Forest
Data type Occurrence records File: oo_717025.xlsx https://binary.pensoft.net/file/717025 Iuri Veríssimo, Beatriz Maria da Silva Jorge

7D6553EE-2139-596B-BF9A-2C317F7D4F1D 10.3897/BDJ.10.e86756.suppl2 Supplementary material 2 SIMPER analysis results Data type average between-group Bray-Curtis dissimilarity Brief description SIMPER analysis results: Contribution of each species to overall dissimilarities amongst sampling areas. File: oo_711742.xlsx https://binary.pensoft.net/file/711742 Cecília Andreazzi

## Figures and Tables

**Table 1.** Sampling sites with camera traps in the Estação Biológica Fiocruz Mata Atlântica, Rio de Janeiro.

**Point**	**Habitat**	**Setting**	**Removal**	**Sampling effort (days)**
A14	Peridomicile	14/01/2019	04/07/2019	171
B13	Peridomicile	30/11/2018	10/07/2019	222
B14	Peridomicile	10/12/2018	10/07/2019	212
B15	Peridomicile	10/12/2018	10/07/2019	212
C13	Peridomicile	27/11/2018	09/01/2019	43
C14	Peridomicile	13/11/2018	04/07/2019	233
D13	Peridomicile	13/11/2018	04/07/2019	233
C12	Transition forest	29/06/2018	16/07/2018	17
D10	Transition forest	29/06/2018	16/07/2018	17
D11	Transition forest	14/01/2019	15/05/2020	285
E09	Transition forest	29/06/2018	16/07/2018	17
E10	Transition forest	16/01/2019	30/08/2019	226
E08	Forest core	11/09/2018	11/10/2018	30
F06	Forest core	11/09/2018	11/10/2018	30
F07	Forest core	11/09/2018	11/10/2018	30
G04	Forest core	24/01/2019	12/08/2019	200
G05	Forest core	24/01/2019	26/11/2019	306
G06	Forest core	11/09/2018	11/10/2018	30
H04	Forest core	24/01/2019	12/07/2019	169

**Table 2.** Mid- and large-sized mammal species recorded by camera-traps (1), interviews (2) and occasional observation (3) in the Estação Biológica Fiocruz Mata Atlântica, Rio de Janeiro, including their conservation status at national (Instituto Chico Mendes de Conservação da Biodiversidade 2018) and global (IUCN 2021) scales (LC = Least Concern, NT = Near Threatened, VU = Vulnerable, EN = Endangered, NE = Not Evaluated). Restrictions on international trade due to degrees of threat follow the Convention on International Trade in Endangered Species of Wild Fauna and Flora (2021): Appendix I (high risk of extinction [AP1]), Appendix II (moderate risk of extinction [AP2]) and Appendix III (species protected in at least one country [AP3]).

**Taxa**	**English name**	**Record**	**Conservation status (IUCN/ICMBio/CITES)**
**Didelphimorphia**			
**Didelphidae**			
*Didelphisaurita*	Big-eared opossum	1, 2, 3	LC / LC / -
**Cingulata**			
**Dasypodidae**			
*Dasypusnovemcinctus*	Nine-banded armadillo	1, 2	LC / LC / -
**Chlamyphoridae**			
*Cabassoustatouay*	Greater naked-tailed armadillo	2	LC / LC / -
*Euphractussexcinctus*	Six-banded armadillo	2	LC / LC / -
**Pilosa**			
**Bradypodidae**			
*Bradypusvariegatus*	Brown-throated sloth	2, 3	LC / LC / AP2
**Myrmecophagidae**			
*Tamanduatetradactyla*	Southern tamandua	1, 2, 3	LC / LC / -
**Primates**			
**Callithrichidae**			
*Callithrix* sp.	Common marmoset	2, 3	Hybrid
*Leontopithecusrosalia*	Golden lion tamarin	2, 3	EN / EN / AP2
**Cebidae**			
*Sapajusnigritus*	Black capuchin	2, 3	NT / - / -
**Carnivora**			
**Canidae**			
*Cerdocyonthous*	Crab-eating fox	1, 2	LC / LC / AP2
*Canislupusfamiliaris*	Domestic dog	1	Domestic
**Procyonidae**			
*Nasuanasua*	Coati	1, 2, 3	LC / LC / -
*Procyoncancrivorus*	Crab-eating raccoon	1, 2	LC / LC / -
**Felidae**			
*Leopardusguttulus*	Southern little spotted cat	1, 2	VU / VU / AP1
*Feliscatus*	Domestic cat	1	Domestic
**Rodentia**			
**Erethizontidae**			
*Coendouspinosus*	Paraguayan hairy dwarf porcupine	2, 3	LC / LC / LC
**Cuniculidae**			
*Cuniculuspaca*	Lowland paca	1, 2	LC / LC / AP3
**Dasyproctidae**			
*Dasyproctaleporina*	Red-rumped agouti	1, 2	LC / LC / -
**Lagomorpha**			
**Leporidae**			
*Sylvilagustapetillus*	Coastal tapeti	1, 2	VU / NE / -

**Table 3.** Absolute records (left) and camera-day records (right) of mid- and large-sized mammal species recorded by camera-traps per sampling area in the Estação Biológica Fiocruz Mata Atlântica, Rio de Janeiro.

Species	Peridomicile		Transition		Forest Core		Total	
*C.l.familiaris*	122	0.092	21	0.037	31	0.039	174	0.065
*C.thous*	30	0.023	10	0.018	–	0.000	40	0.015
*C.paca*	162	0.122	6	0.011	111	0.140	279	0.104
*D.leporina*	1	0.001	66	0.117	97	0.122	164	0.061
*D.novemcinctus*	11	0.008	5	0.009	26	0.033	42	0.016
*D.aurita*	146	0.110	166	0.295	58	0.073	370	0.138
*F.catus*	21	0.016	–	0.000	–	0.000	21	0.008
*L.guttulus*	–	0.000	5	0.009	5	0.006	10	0.004
*N.nasua*	–	0.000	2	0.004	16	0.020	18	0.007
*P.cancrivorus*	–	0.007	10	0.018	23	0.029	42	0.016
*S.tapetillus*	8	0.006	–	0.000	–	0.000	8	0.003
*T.tetradactyla*	9	0.007	4	0.007	8	0.010	21	0.008
Total	519	0.391	295	0.525	375	0.472	1.189	0.443

**Table 4.** Distribution of mid- and large-sized mammal species recorded by camera-trap in the Estação Biológica Fiocruz Mata Atlântica, Rio de Janeiro.

Species	Peridomicile							Transition					Forest core						
	A14	B13	B14	B15	C13	C14	D13	C12	D10	D11	E09	E10	E08	F06	F07	G04	G05	G06	H04
*C.l.familiaris*	16	17	49	8	5	22	5	–	–	9	–	12	–	–	2	–	5	–	24
*C.thous*	1	4	7	1	17	–	–	–	–	10	–	–	–	–	–	–	–	–	–
*C.paca*	–	56	–	–	–	3	103	–	–	4	–	2	–	–	–	31	44	–	36
*D.leporina*	–	–	–	1	–	–	–	–	1	51	–	14	1	2	–	35	39	–	20
*D.novemcinctus*	8	2	–	1	–	–	–	–	–	3	–	2	–	–	–	15	10	–	1
*D.aurita*	24	30	36	23	–	2	31	6	5	51	5	99	6	6	–	34	7	1	4
*F.catus*	3	7	2	1	2	3	3	–	–	–	–	–	–	–	–	–	–	–	–
*L.guttulus*	–	–	–	–	–	–	–	–	–	3	–	2	–	–	–	–	3	–	2
*N.nasua*	–	–	–	–	–	–	–	–	–	–	–	2	3	1	1	2	5	1	3
*P.cancrivorus*	–	5	1	–	–	2	1	–	–	7	–	3	–	–	–	19	2	–	2
*S.tapetillus*	–	2	–	–	–	–	6	–	–	–	–	–	–	–	–	–	–	–	–
*T.tetradactyla*	2	2	1	3	1	–	–	–	–	2	–	2	–	–	–	2	4	1	1
